# Extending the translational science benefits model to implementation science for cancer prevention and control

**DOI:** 10.1017/cts.2024.582

**Published:** 2024-11-05

**Authors:** Karen M. Emmons, Ross C. Brownson, Douglas A. Luke

**Affiliations:** 1 Harvard T.H Chan School of Public Health, Boston, MA, USA; 2 Prevention Research Center, Brown School at Washington University, St Louis, MO, USA; 3 Department of Surgery, Division of Public Health Sciences and Alvin J, Siteman Cancer Center, Washington University School of Medicine, Washington University, St Louis, MO, USA; 4 Center for Public Health Systems Science, Washington University, St Louis, MO, USA

**Keywords:** Implementation science, translational benefits, cancer prevention and control

## Abstract

**Introduction:**

There is increasing pressure on the federal research budget and shifting public opinions about the value of the academic enterprise. We must develop and apply metrics that demonstrate the broad benefits of research for health and society. The Translational Science Benefits Model (TSBM) measures the impact of large-scale translational science initiatives, such as the National Cancer Institute’s Cancer Moonshot. TSBM provides the scaffolding to illustrate how science has real-world health impacts. We propose an expansion of the TSBM to explicitly include implementation-focused outcomes.

**Methods:**

TSBM includes four categories of benefits, including (1) clinical and medical, (2) community and public health, (3) economic, and (4) policy and legislative. Implementation science outcomes serve as a precursor to the model’s established domains of impact and can help to sharpen focus on the translational steps needed to achieve a broad range of impacts. We provide several examples of studies that illustrate these implementation outcomes and other clinical and community benefits.

**Conclusions:**

It is important to consider a broad range of scientific impacts and the conditions that are necessary to achieve them. The expansion of the TSBM to include implementation science outcomes may help to accelerate the cancer community’s ability to achieve the goal of preventing 4 million cancer deaths by 2047.

## The importance of research impact

In the last several years, there has been a growing emphasis on better understanding and articulating the societal benefits of the nation’s investment in scientific research. Although some research leads to outcomes with clear and large benefits, such as vaccine development [1,2], for much of scientific research the benefits are less clear with longer time frames or are more aspirational. The National Institutes of Health (NIH) review process assesses the potential impact, and most investigators no doubt want their scientific passion to be reflected in societal impact, even as they recognize impact as essential to their career development. However, without a framework for considering the benefits of their work, scientists have been largely focused on metrics that have meaning within their academic institutions and are associated with academic advancement (e.g., grant submissions, grant funding, publications, and citations) rather than outcomes that make an impact for the public at large. With increased pressure on the federal budget and subsequent downward pressure on research funding, as well as shifting public opinions about the value of the academic enterprise, it is past time that we apply metrics that can demonstrate the broader benefits of research for human health and society at large.

Since 2014, the United Kingdom has utilized the Research Excellence Framework (REF) to evaluate universities with regard to the impact of their respective research portfolios [3]. The goals of the REF are to provide accountability for public investment in university research programs and to evaluate the benefits of this investment. In addition, the REF is used to guide funding decisions across the UK’s nationally funded university system. The REF includes three elements: (1) the quality of outputs (e.g., publications, performances, and exhibitions), reflecting more traditional academic metrics, (2) impact beyond academia, and (3) the environment that supports research. The REF is assessed at an institutional level, reflecting both the research environment and the collective productivity and impact of the members of the institution.

Although there is no direct parallel to the REF in the USA, there has been increasing emphasis on assessing the impact of specific scientific research efforts. The Translational Science Benefits Model (TSBM) was developed by the Institute for Clinical and Translational Sciences Tracking and Evaluation Team at Washington University in St Louis as a guiding framework for measuring the impacts of large-scale translational science initiatives. The TSBM [4] was developed to provide the scaffolding by which investigators and institutions could identify the ways in which their science had real-world clinical and community health impacts. TSBM is typically assessed at the research project level, and specifies four categories of health and societal benefits, including (1) *clinical and medical benefits*, such as the adoption and implementation of new tools and procedures in clinical settings as an outcome of clinical and translational research; (2) *community and public health benefits* that enhance health care or community and population well-being; these benefits can range from the specific services provided (e.g., on-site cancer screening, integrated behavioral health) to the characteristics of the care provided (e.g., accessibility, quality); (3) *economic benefits*, including financial improvements, improved cost-effectiveness of treatments, and new commercial entities resulting from clinical and translational research, and (4) *policy and legislative benefits*, such as formal adoption of scientific evidence into organizational or public policies, legislation, or governmental standards based on clinical and translational research. Each of these domains includes a number of benefit indicators, representing specific new or improved benefits that have accrued from research.

## Research impact in implementation science and cancer control

The TSBM has begun to receive significant attention in the translational research community [5]. The National Center for Advancing Translational Science placed significant focus on measuring the impact of translational science in its most recent funding announcement (PAR 21-293) for translational science hubs (CTSAs). Many CTSAs are drawing upon TSBM to guide their evaluation of broad scientific impact. A parallel activity within the CTSA program has been the development of the Integrative Framework of Dissemination, Implementation, and Translation [6], which presents a coherent set of dissemination and implementation science strategies and methods to accelerate research translation, in addition to traditional translational research methods. Implementation science is a field that focuses on increasing the use of scientific evidence in everyday practice and policy. Implementation science has also been given a more prominent role in the CTSAs, with a requirement in the current funding announcement for all centers to engage in dissemination and implementation (D&I) activities to support innovative approaches to translational research.

There are other large-scale scientific initiatives that have a significant potential for broad impact. For example, the National Cancer Institute’s (NCI) multi-billion dollar Cancer Moonshot Initiative, launched in 2016 by the Obama administration, aimed to dramatically accelerate the pace of cancer research [7]. In its first 5 years, Cancer Moonshot focused on accelerating discovery, increasing collaboration, and expanding data sharing among the research community. Over 250 research projects have been supported through Moonshot funding. A second phase of the Cancer Moonshot Initiative was announced in 2022 by the Biden administration, with the bold goal of reducing the cancer mortality rate by half within 25 years. The relaunched Initiative puts particular emphasis on the collaboration needed across federal agencies in order to achieve this goal. Many of the efforts funded relate to new approaches that should, over time, yield significant and direct impact on the American public, such as the network to facilitate direct patient engagement in tumor profiling and the Adult Immunotherapy Network. To our knowledge, there has not been an effort to apply a common evaluation model to this Initiative.

The Cancer Moonshot^SM^ Initiative has also included a significant focus on the role of implementation science in cancer research, particularly related to expanding the use of proven cancer prevention and early detection strategies. [8] The Moonshot^SM^-funded Implementation Science Centers in Cancer Control Network (ISC3) was created to allow for efficient and equitable translation of evidence-based approaches to reduce cancer risk and improve outcomes [9]. ISC3 includes 7 P50 Centers that have research-practice partnerships at their core and are designed to create the opportunity for a series of pilot studies to explore new and sometimes risky ideas and embed in their infrastructure a two-way engagement and collaboration essential to stimulating lasting change. All of the centers included a strong interest in maximizing impact in ways that align well with the TSBM.

Given the scale of the investment and its ability to change the face of cancer in the USA, it will be important to consider the impact of the investment in Cancer Moonshot through a broad range of common metrics for measuring the downstream outcomes of all relevant research, including implementation science studies. Given the distinct nature of implementation science methodologies and outcomes and the increased emphasis on dissemination and implementation science in Cancer Moonshot and the CTSA Consortium, we determined that there would be value in expanding the TSBM to explicitly include implementation-focused outcomes.

The lead investigators in the ISC3 Network undertook a process of articulating a range of benefits of implementation science that extend beyond traditional academic metrics. We first conducted an expert review/consensus process in which a small group of senior leaders in the field developed a set of candidate outcomes. A detailed review and substantial input process were then conducted with leaders from the seven ISC3 centers and NCI implementation science experts. The expanded model was then finalized through a second consensus process.

**Figure 1.**
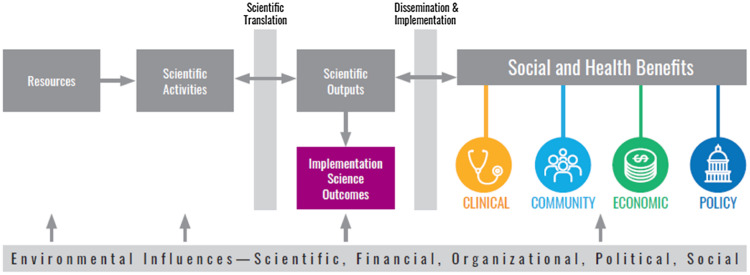
Logic model showing expansion of translational science benefits model to include implementation science outcomes. *Adapted from Luke et al., 2018*.

Implementation outcomes include conceptually distinct features of the implementation process, including acceptability, adoption, appropriateness, feasibility, fidelity, implementation cost, penetration, and sustainability [10]. A recent scoping review found that acceptability, feasibility, and fidelity are the most commonly reported implementation outcomes, with considerably fewer studies examining the remaining outcomes [11]. Implementation outcomes reflect the specific parameters of an implementation effort that are associated with its effectiveness. Our goal was to integrate these types of implementation outcomes in a new TSBM outcomes domain that captures implementation science disciplinary impact. Figure 1 illustrates the original TSBM model with the expanded “Implementation Science Outcomes” box added to the model. Implementation science outcomes serve as a precursor to the model’s four established domains of health and social impact. These implementation science outcomes are organized into two sub-categories, reflecting areas that have been identified as having significant potential for impact [9,12]. First, methods and measures refer to the outcomes of specific efforts to design and measure the conditions needed for broader impact. For example, community benefits will be more likely if evidence-based interventions can be effectively adapted to a broad range of community settings and the impact of those adaptations can be effectively measured.

The second sub-category, capacity building, is the development of the resources needed to yield clinical, community, economic, and policy benefits. For example, in order for research to yield clinical benefits, there has to be engagement of the clinical teams and institutions that will use the evidence and integrate it into practice. Researchers should also have the capacity to work with partners to increase their use of evidence, which decades of slow uptake of scientific findings have aptly demonstrated. Implementation science plays a critical role in enhancing the uptake of scientific evidence in practice and policy. Without efforts to actually use the science that we have produced, the impact will be minimized. Including implementation science-related outcomes as part of TSBM will help to sharpen the field’s focus on this critical translational step. Table 1 presents specifications of the range of indicators that could be considered.

**Table 1.** Implementation science domain of the translational science benefits model

Implementation Science Methods and Measures
**Benefit Indicators**
*Measures development* New measures of implementation determinants, processes, or outcomes
*Methods development* New methods for implementation strategy selection and optimization, or for identifying and prioritizing implementation determinants New methods and toolkits for identifying and applying theories, models, and frameworks (TMFs) focused on health equity Comprehensive summaries of existing methods and make recommendations for improvements New engagement or co-creation strategies Pragmatic costing tools to inform decision-makers and implementation science (IS) researchers New methods for assessing implementation and setting context Identify gaps in the literature
*Use of rapid cycle/data collection strategies* Rapid needs assessment Used rapid cycle testing design
*Adaptation* Developed pragmatic, low-burden approaches to measuring adaptation, fidelity, and implementation cost Technological tools for tracking adaptations in clinical and community settings Function focused fidelity scales that are easy to administer in clinical and community settings Develop or adapt an implementation process or strategy with an explicit focus on health equity
*Develop methods for examining clinical/community partner data in new ways/formats that support their work*
***Capacity Building***
*Building partner/practitioner research capacity* Partners lead or participate on grants, publications, presentations Develop partner’s own research infrastructure Develop partner skills in implementation processes Develop practitioner toolkits for integrating equity and/or costing into IS Develop tools to encourage the iterative use of IS frameworks for implementation, adaptation, and sustainment of evidence-based programs in practice Develop tools to encourage the iterative use of IS frameworks by partners
*Engagement* Develop a relevant and actionable strategy for the return of results to research partners Include partner in the selection of pilot grants Increase diversity of engaged partners
*Build IS research capacity* Include early investigators, trainees Increase diversity of investigator teams Increase skills of mentors Increase skills of early investigators and trainees at all levels Develop investigator toolkits for integrating equity into implementation science Extend IS efforts in the context of the partnership to new content areas in cancer control (e.g., climate change) Develop/refine tools to aid in the planning of IS projects, selection, combination, adaptation, use, and assessment of IS TMFs

## Examples of implementation outcomes that are precursors to translational benefits

**Figure 2.**
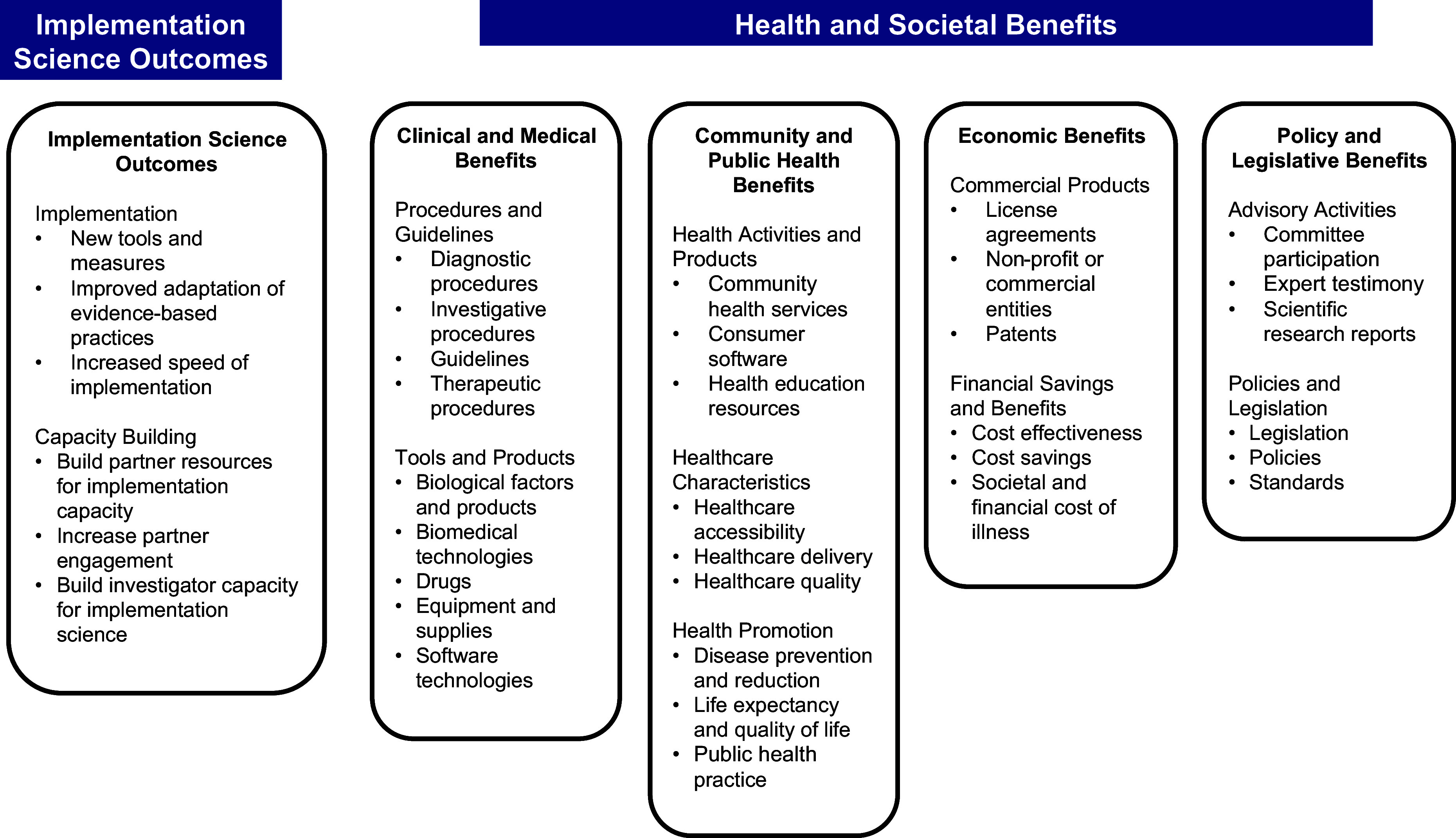
Expanded translational science benefits model with implementation science considerations.

We draw on the work of the ISC3 Centers, as well as other literature, to provide examples of the role of implementation science disciplinary outcomes in ensuring translational benefits of research. There are a limited number of published examples in the cancer control literature at the moment, but we anticipate that will change over time. The types of outcomes we consider fall into one of two disciplinary advances: (1) implementation science methodologies and (2) disciplinary capacity building (see Fig. 2).

Some examples of the ISC3 Network’s impact applied to TSBM *methods-related implementation outcomes* include the development of a method for adapting evidence-based interventions to increase a focus on equity outcomes. Aschbrenner and colleagues developed the Stakeholder and Equity Data-Driven Implementation (SEDDI) process to facilitate using healthcare data to identify patient groups experiencing gaps in the use of evidence-based interventions (EBIs) and to rapidly adapt EBIs to achieve greater access and equitable outcomes [13]. Through another NCI Moonshot^SM^ program, Menon and colleagues used an adaptation framework and cultural consultations with the community and clinicians to adapt a tailored colorectal cancer screening navigation program for the American Indian Community [14].

Other examples of measures and method development that were conducted outside of Cancer Moonshot^SM^ include the application of budget impact analysis to implementation decision-making and planning [15] and the development of psychometrically strong, pragmatic measures of implementation mechanisms to improve the field’s understanding of the active ingredients of implementation success [16]. The consistent utilization of pragmatic measures of key implementation outcomes [17] across network studies has increased the standardization of outcomes assessment in real-world settings. There has also been considerable work to adapt and enhance existing theories, models, and frameworks to more fully address the context for implementation, including a more explicit focus on health equity [18]. The ISC3 Network has further developed approaches to participatory logic model development, which is a key strategy to engage stakeholders in developing evaluation plans that can enable impact at clinical, community, or other levels [19]. The Moonshot ^SM^-funded Cancer Center Cessation Initiative (C3I) used data envelopment analysis to evaluate the efficiency with which evidence-based tobacco treatment programs were used at participating cancer centers [20]. A final example comes from recent work by Asada and colleagues to apply hybrid design methodology to policy studies, which may facilitate work that leads to policy impact [21].

There have also been some excellent examples of *capacity building* as an implementation outcome, including the adoption of research policies that ensure that community partners on implementation studies are always included as coauthors on manuscripts resulting from an implementation partnership [22]. The ISC3 Network also utilized social network analysis to identify opportunities for further engaging and building the capacity of more marginalized members [23]. Outside of Cancer Moonshot^SM^, Goodman and colleagues developed the Research Engagement Survey Tool to assess the level of partner engagement in implementation and other types of research studies [24], enabling researchers in the ISC3 Network to understand and improve their engagement of key stakeholders in efforts to implement evidence-based interventions. The benefits of mentored training programs for building research capacity in implementation science have also been identified and have been a significant component of the ISC3 network activities, both through the Implementation Science Scholars Program at Washington University in St Louis [25] and NCI’s Consortium on Cancer Control Implementation Science [26], which the ISC3 Network supports. The ISC3 working group considers the expanded TSBM as part of its evaluation framework for network activities, and the network members have also committed to continuing an emphasis on assessing translational impacts. Several network centers are planning to integrate this approach into other ongoing work.

Several areas of emphasis and new directions will enhance the use and usefulness of the TSBM [27]. First, a set of planning and evaluation tools have been developed to help guide TSBM users as they plan, monitor, and demonstrate the impacts of their research. Second, equity is an increasingly important area for the implementation science and cancer control [28]. With the help of community partners, the TSBM is being expanded to include a more explicit focus on equity. This development work is in progress, but after community input, the TSBM is being expanded to include more emphasis on community capacity building, equitable delivery of healthcare innovations to all, and greater access to economic resources and power, among others.

Finally, a more complete application of the TSBM will require actions across at least three levels (i.e., individual researchers, academic institutions, funding agencies). Researchers can be more intentional in tracking impacts and telling the story of their research. For example, including team members with skills in journalism can provide depth in storytelling that researchers often lack. Community partners are also typically more skilled at telling the story of their work than researchers. Academic institutions can build real-world impact criteria in promotion and tenure guidelines. Funders can require dissemination and sustainment plans that focus on audiences outside of academia. The TSBM indicators can also provide funders and policy audiences with a useful set of intermediate endpoints on the pathway to long-term return on investment. We need to more effectively communicate the incremental process of research resulting in long-term dividends.

## Conclusions

The TSBM has been an extremely valuable tool for considering the broader societal impacts of research, pushing investigators to think beyond traditional academic metrics to the value that their science can bring to society. By expanding the model to include implementation science outcomes as precursors to these societal benefits, we hope to unpack the process by which research could more efficiently and effectively have an impact. We provide examples of the range of impacts that are reflected in the expanded TSBM and hope that future evaluations of individual studies and large initiatives will use similar approaches. The added perspective on implementation science can also serve to ensure that equity is considered in ways that increase the likelihood that broader impacts of science are fairly distributed and do not cause unintended harm.
